# Noncontact friction via capillary shear interaction at nanoscale

**DOI:** 10.1038/ncomms8359

**Published:** 2015-06-12

**Authors:** Manhee Lee, Bongsu Kim, Jongwoo Kim, Wonho Jhe

**Affiliations:** 1 Department of Physics and Astronomy, Institute of Applied Physics and Centre for THz-Bio Application Systems, Seoul National University, Seoul 151-747, Korea; 2 Present address: Samsung Electronics, Giheung Campus, Yongin 446-920, Korea

## Abstract

Friction in an ambient condition involves highly nonlinear interactions of capillary force, induced by the capillary-condensed water nanobridges between contact or noncontact asperities of two sliding surfaces. Since the real contact area of sliding solids is much smaller than the apparent contact area, the nanobridges formed on the distant asperities can contribute significantly to the overall friction. Therefore, it is essential to understand how the water nanobridges mediate the ‘noncontact' friction, which helps narrow the gap between our knowledge of friction on the microscopic and macroscopic scales. Here we show, by using noncontact dynamic force spectroscopy, the single capillary bridge generates noncontact friction via its shear interaction. The pinning–depinning dynamics of the nanobridge's contact line produces nonviscous damping, which occurs even without normal load and dominates the capillary-induced hydrodynamic damping. The novel nanofriction mechanism may provide a deeper microscopic view of macroscopic friction in air where numerous asperities exist.

## Abstract (web summary)

The contribution from water bridges at nanoscale between rough surfaces is important for macroscopic friction under ambient conditions. Here, Lee *et al*. show that water nanobridge produce noncontact friction originated from the pinning–depinning dynamics of the contact line at the interface.

## Graphical abstract

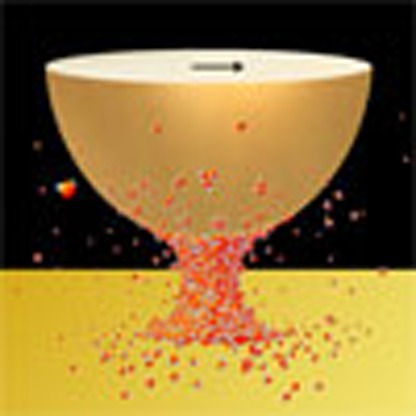

Friction is the force against sliding between two bodies in contact and the phenomenon spans several orders of magnitude from the nanometre scales of biological molecules and atomic contacts^1^,^2^,^3^ to the kilometre scales of earthquakes^4^, having scientific as well as technological importance^5^. In dry condition, the dominant microscopic mechanism for the contact friction is the nonlinear, thermally activated stick-slip dynamics of the contacting asperities, which increases the friction force logarithmically with the sliding velocity^6^,^7^. Recent studies have shown that there exists even the minute, noncontact energy dissipation at a nanometric separation due to the fluctuating electromagnetic fields^8^. Therefore, the contact-sliding friction in dry condition seems to be largely a matter of interactions between the objects at the direct contact interface.

In ambient condition, however, there always exists a third object between contact bodies, the capillary–condensed water bridge or meniscus^9^, which binds two nanoscale asperities and thereby strongly affects friction^10^,^11^. Such a humidity-assisted friction has been understood as associated with the thermally activated capillary condensation of water vapour into liquid water that occurs between hydrophilic surfaces in air^12^,^13^,^14^. For the contact sliding of a single asperity, the capillary-mediated friction decreases logarithmically with the velocity due to the proportionally diminishing bridge size and capillary attraction^15^,^16^. On the other hand, the water bridge can be also formed in the nanometric gap between two distant asperities^17^, with its nanometric height maintained constant, and thus results in the noncontact friction, unlike in dry condition, even in the absence of normal load on the solid–solid contact.

The liquid bridge has been usually considered to add an additional normal load, increasing the microscopic real contact area and thereby enhancing the overall friction^10^,^11^,^18^. However, such an indirect effect does not describe the friction that the bridges may produce directly between contact as well as noncontact nano-asperities. This direct contribution is important, in particular, not only because the distant asperities consist of the large part of the apparent contact area beyond the real contact area (Fig. 1a)^19^,^20^ but also because the water bridge can be easily formed in the gap of distant asperities with separation less than ∼10 nm in ambient condition^21^ (see also Supplementary Note 1). Although the noncontact friction mediated by a single water bridge is about 10% of contact friction due to a single asperity, as we shall show later, the overall contribution of noncontact asperities can be significant because they usually exist numerously due to the multi-asperity feature at the macroscopic contact interface. Therefore, for a better understanding of friction in air, it is critical to address the noncontact sliding friction that the single capillary bridge mediates, which helps the gap in our understanding of the microscopic and macroscopic scale friction.

Here we study theoretically and experimentally the capillary ‘shear' interaction of a single water nanobridge (Fig. 1b) that produces the noncontact sliding friction, by using a shear-mode noncontact atomic force microscope (AFM)^17^,^22^. The unique features of our approach include the following. First, the pure effects of the water bridge are measured by noncontact operation. Second, the velocity dependences are studied for the constant bridge volumes. Finally, quantitative description of the noncontact friction is made in terms of shear modulus, which enables quantitative comparison with the contact friction. We show that the bridge-mediated noncontact interaction produces the solid-like elasticity, as well as the novel nonviscous damping that originates from the molecular-scale pinning–depinning dynamics of the tip–bridge contact line.

## Results

### Formation and detection of a nanosized water bridge

The noncontact AFM employs the quartz tuning fork oscillator, with a sharp tip attached, which has high stiffness (∼10^4^ N m^−1^) and a high quality factor (∼10^3^). Consequently, the system not only realizes the stable formation of the single capillary nanobridge with controlled height and volume^17^, avoiding the jump-to-contact instability typically experienced in soft cantilever-based AFM, but also allows sensitive measurement^23^ and quantitative analysis^24^ of the associated interactions. When the laterally oscillating tip approaches the mica substrate, a water nanobrdige is produced by capillary condensation in the tip–sample nanometric gap (Methods), causing a change in the measured quantities, the amplitude and phase of the tip oscillation. Once the nanobridge formation is observed, the tip retracts to avoid the tip–substrate hard contact, while the amplitude change and phase shift are recorded until the bridge is ruptured. The measured experimental data are analysed using the dynamic force spectroscopy (DFS) method^25^ to obtain quantitatively the associated bridge-mediated interactions.

### Nanosized water bridge-mediated shear interaction

**Figure 1:**
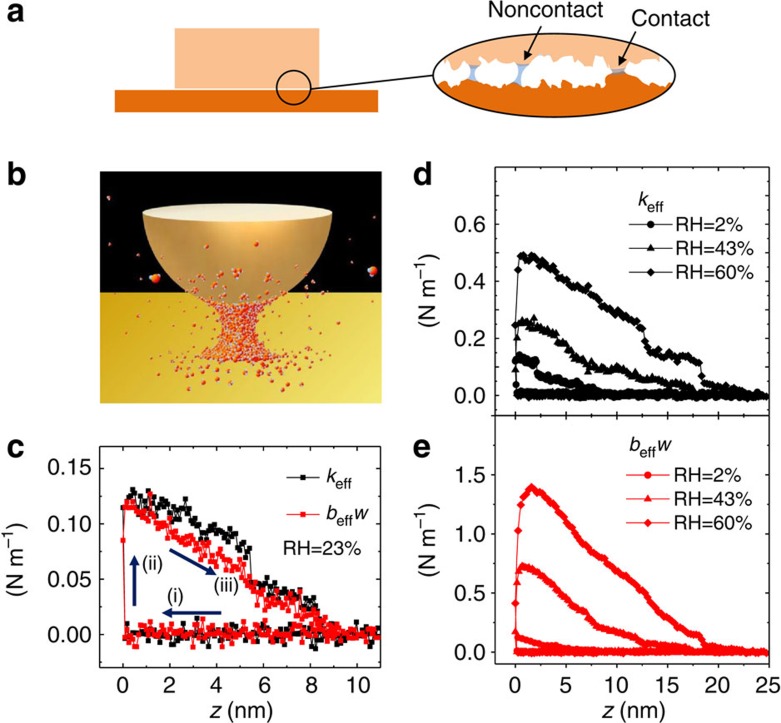
Noncontact sliding friction resulting from capillary shear interaction. (**a**) Schematics of the microscopic interface between two sliding macroscopic bodies in contact in ambient condition. Within the nanoscale gap, numerous water nanobridges are formed by capillary condensation between two noncontact as well as contact asperities, and they produce the bridge-induced resistive force besides the solid–solid contact friction. (**b**) Experimental scheme of measuring the bridge-mediated noncontact friction. We used a homebuilt noncontact atomic force microscope that allows stable and controllable formation of the capillary water nanobrdige in the gap between the gold tip and mica substrate, and measures precisely the associated interaction forces. (**c**) Typical elastic *k*_eff_ and damping *b*_eff_*w* coefficients as a function of the tip–sample distance *z* during (i) tip approach→(ii) capillary formation (at *z*=0)→(iii) tip retraction. (**d**,**e**) The force constants *k*_eff_ and *b*_eff_*w* obtained at different relative humidity (RH). The bigger bridge (having the longer rupture distance) is formed at the higher RH (circles→triangles→diamonds). Here the tip–sample contact point is estimated as the position *z*≈−4.6 nm (see Supplementary Note 2 for details). Notice that in **c**–**e**, the elasticity shows noticeable discrete-decrease behaviours, unlike the damping, during tip retraction before rupture, which may be attributed to the corresponding discrete decrease of the length (or the radius 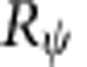) of the three-phase contact line at the tip–bridge interface under the constant volume condition, as first observed in ref. 17.

The quartz tuning fork-based DFS is ideal for our goal, because it allows high measurement sensitivity for the stiff mechanical oscillator and detects simultaneously and separately the conservative *F*_*k*_ and nonconservative *F*_*b*_ components of the total tip–liquid interaction force *F*_int_ (=*F*_*k*_+*F*_*b*_). Furthermore, by this technique, a wide sliding-velocity range is possible while the bridge volume is maintained constant. Specifically, this technique allows one to obtain ‘effective' elastic *k*_eff_ and damping *b*_eff_ coefficients. The respective forces are then derived as *F*_*k*_≈−*k*_eff_*x* and 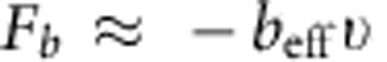, where *x* is the lateral displacement of the tip and *v* is the corresponding shear velocity^22^,^24^. Figure 1c shows the typically measured elasticity *k*_eff_ and *b*_eff_*w* (here, *w* is the angular frequency of tip oscillation), and they exhibit an abrupt jump when the capillary condensation occurrs, which defines the position *z*=0. The interaction constants *k*_eff_ and *b*_eff_*w* increase with the relative humidity (RH) or the rupture distance (that is, volume) of the bridge, as shown in Fig. 1d,e. Since *v*=0 when *x*=*A* (and also *v*=*wA* when *x*=0) for the harmonic motion of the tip (with its oscillation amplitude *A*), we can measure separately two instantaneous peak values of the oscillatory interaction force, *F*_*k*_ and *F*_*b*_, at *x*=*A* and *x*=0, respectively; *F*_int_=*F*_*k*_≈−*k*_eff_*A* at *x*=*A* and *F*_int_=*F*_*b*_≈−*b*_eff_*wA* at *x*=0.

### Mechanism of water bridge-mediated noncontact friction

**Figure 2:**
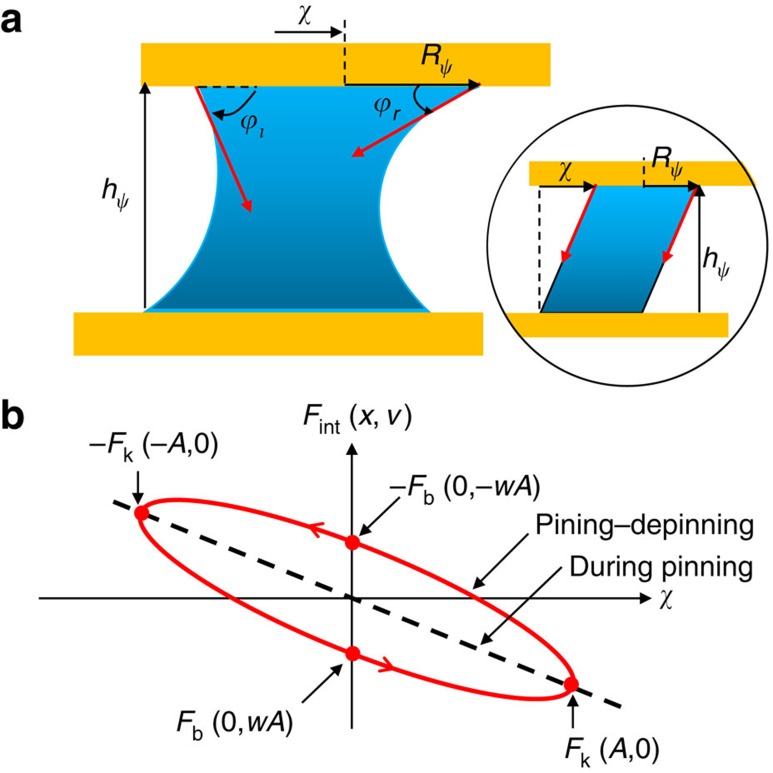
Contact-line-induced capillary shear interaction. (**a**) Schematics of the bridge under shear, where the three-phase contact line at the tip–liquid interface induces the restoring elastic force as well as the nonviscous dissipative force due to the contact-line dynamics at the molecular scale. Inset shows an imaginary cylindrical column of the bridge for a simple estimate of its elasticity. (**b**) Characteristic representation of the contact-line-induced shear force. While the bridge with its contact line pinned exhibits the elastic response (dashed line), the moving contact line induces the dissipative force associated with its pinning–depinning kinetics and splits the force–distance curve depending on the moving direction (red curve), resulting in the force hysteresis. The area of the hysteresis represents the dissipated energy associated with the nonviscous damping *b*_eff_*w*, whereas the tilt of the hysteresis is the shear elasticity *k*_eff_.

Now, let us discuss the microscopic mechanisms of the liquid bridge-mediated shear interaction. At first, we show that the measured elastic and damping properties cannot be explained by the hydrodynamic interaction or capillary negative pressure exerted on the tip–bridge interfacial area *σ*. The hydrodynamic viscous drag causes dissipative damping on the shearing interfacial area under the usual assumption of no-slip boundary condition, and the corresponding damping coefficient, when expressed in the same unit of the elasticity, is given by 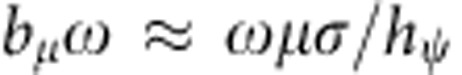, where *μ* is the viscosity of water and 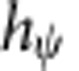 the bridge height (Fig. 2a). While there have been reports on the substantial enhancement of *μ* under subnano-confinement, the present confining height 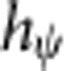 is much longer (>4.5 nm). Therefore, using the bulk viscosity of water^26^,^27^,^28^ and the relevant quantities, *σ*=*π*(15 nm)^2^ and 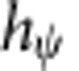
=6 nm, which can be obtained by using the theoretical formalism and estimation procedure of the tip–sample distance developed in detail in the Supplementary Notes 2 and 3, we obtain 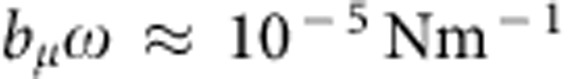, 10^5^ times smaller than the measured value of about 1 N m^−1^. Moreover, the increased viscous damping that occurs near the contact line alters the contact angle as 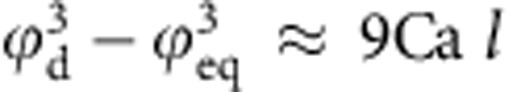, where 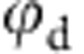 is the dynamically varying contact angle, 
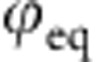
 the equilibrium contact angle, Ca the capillary number and *l* is a logarithmic factor^29^. For the highest *l*∼100, the angle change is at most a few 10^−3^ rad, which is still 10^4^ times less than the measurement (Supplementary Note 4). In addition, since the negative pressure of the bridge exerts an attractive force in normal direction to the tip surface, its lateral shear contribution is also negligible.

As an alternative interaction mechanism, we now demonstrate that the contact line of the tip–bridge interface can generate simultaneously bridge elasticity as well as nonviscous dissipation. First, to derive the elastic component of the capillary shear force, let us consider simply a cylindrical column of water, whose contact line is pinned on the sliding flat tip surface (inset of Fig. 2a) (refer to Supplementary Note 5 for a general round tip). The surface energy *U*_*l*_ with respect to the shear displacement *x* can be exactly solved as, 
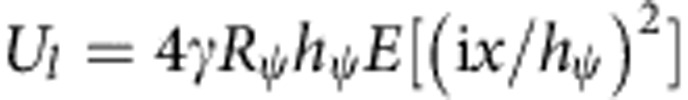
, where 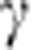 is the surface tension of water, 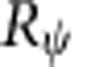 the radius of the contact line and 
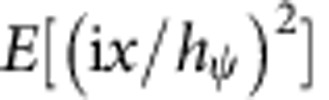
 is the complete elliptic integral of the second kind (see Supplementary Note 6 for derivation). The energy gradient then gives the maximum value of elastic force at *x*=*A*,


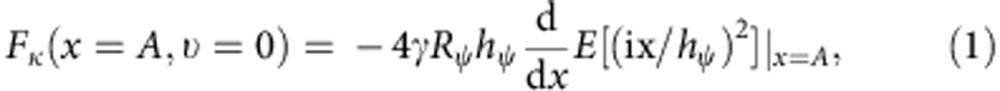


and 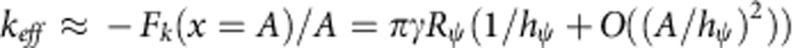. With the parameters, 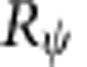
=15 nm and 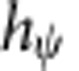
=6 nm (see Supplementary Notes 2 and 3), we find *k*_eff_≈0.6 N m^−1^ in excellent agreement with the experimental results (Fig. 1d).

Besides the pinning of the contact line responsible for the elasticity, however, there can also occur the depinning following each pinning. The pinning–depinning (or stick-slip) kinetics of the contact line induces an energy dissipation and is described by the ‘molecular kinetic theory'^30^. Essentially, the theory provides a relation between the contact-line velocity *V* (relative to the tip surface) and the applied force per unit length on the contact line 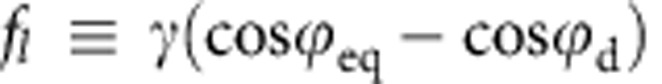, given by 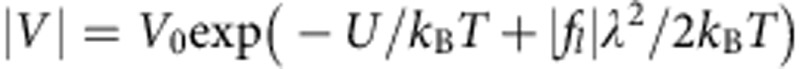, where 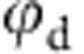 is the dynamic contact angle on the moving contact line. Here this nonlinear pinning–depinning dynamics includes two intrinsic parameters, the length of activated jump *λ* and the activation energy *U* of the jump. The scale factor *V*_0_ is given by *V*_0_≡*λk*_B_*T*/*h*, where *k*_B_ is the Boltzmann constant and *h* the Plank constant.

For a given *U* and *λ*, we can then derive the shear damping force *F*_*b*_ as a function of *V* by summing up *f*_*l*_ around the contact line. The larger tip motion (or higher *v*=*wA*) induces the larger contact-line force, and the larger imposed force triggers more pinning–depinning processes at the contact line^30^,^31^. This microscopic mechanism leads to the overall increase of the velocity *V* along the direction of the exerted force. We thus conjecture that *V*→*V*_0_exp(–*U*/*k*_B_*T*)≡*V*_*i*_ for *A*→0 (that is, no external perturbation) and *V*→*wA* for *A*→∞ (that is, infinitely fast and long travel of the contact line) at the tip position *x*=0 (or at the tip velocity is *wA*), where the bridge experiences a negligible elastic restoring force (equation (1)). Then, introducing an empirical parameter 0≤*α*(*A*)≤1, the degree of slip, we can construct a linear relation between *V* and *A*, as *V=V*_*i*_+*αwA*, where *α*(*A*=0)=0 and *α*(*A*→∞)=1. With this relation, we then obtain the maximum frictional force *F*_*b*_ at *x*=0 and *v*>0 as a function of *A* (Supplementary Note 5),


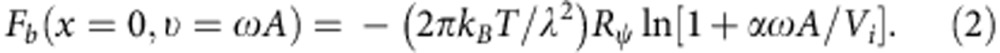


Here we find *F*_*b*_ is proportional to 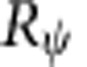 and exhibits the logarithmic dependence on the tip velocity *wA*. Notice that for *v*<0, 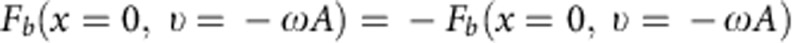
due to the symmetry, resulting in the force hysteresis (Fig. 2b). We also find 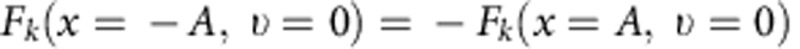.

In summary, the total contact-line-induced shear force is analytically given by the sum of the elastic (equation (1)) and damping (equation (2)) forces, which are our main results. They describe how the contact line of the capillary bridge plays the key role in the shear interaction between two noncontact sliding surfaces: whereas the interfacial energy change due to the liquid-surface elongation during the contact-line pinning produces the restoring force *F*_*k*_ (equation (1)), the microscopic pinning–depinning process of the contact line results in the nonviscous friction *F*_*b*_ (equation (2)). For our oscillatory measurement of the interaction forces, 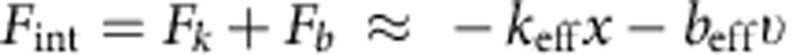, the elastic part *F*_*k*_ contributes to the in-phase component, while the damping part *F*_*b*_ to the out-of-phase component, which are detected separately by the DFS method. Here we note that the pinning–depinning occurs at the contact line, where water molecules hop microscopically over the pinning sites. However, water molecules inside the contact line do not hop under no-slip boundary condition, which is usually assumed between a hydrophilic surface and water. Therefore, because the hydrodynamic viscous damping is 10^4^∼10^5^ times smaller than the measured damping, as discussed earlier, we can consider the experimentally measured interaction originates from the three-phase contact line.

### Noncontact friction via three-phase contact line

**Figure 3:**
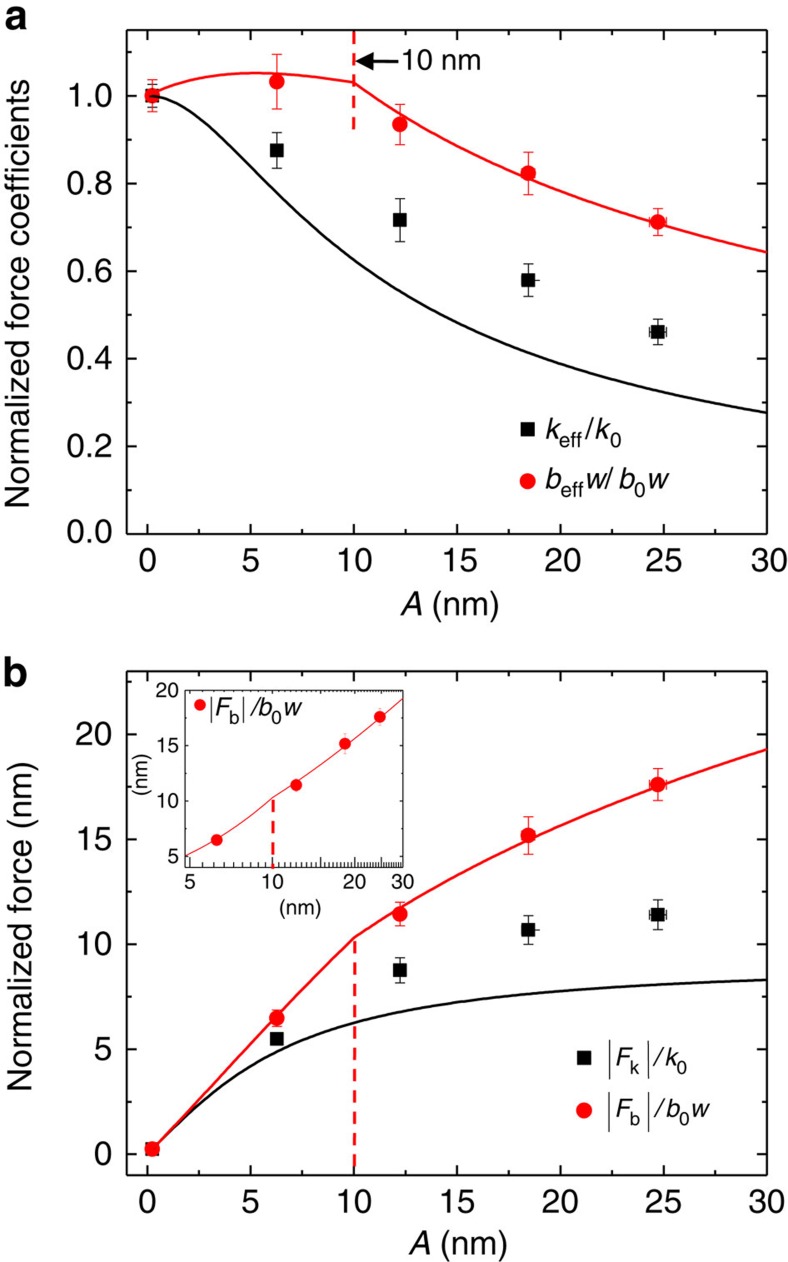
Comparison of the theory of capillary shear interaction with the experimental results. (**a**) Shear-amplitude dependence of *k*_eff_ and *b*_eff_*w*. The normalized *k*_eff_ (black squares) and *b*_eff_*w* (red dots) results are plotted at *z*=1 nm versus *A* for the water bridge formed at RH=30%. The contact-line-induced capillary shear interactions, black (equation (1)) and red (equation (2)) curves, reproduce well the experimental results (black squares and red dots). (**b**) Shear-amplitude (or shear-velocity) dependence of |*F*_*k*_| and |*F*_*b*_|. The inset shows the logarithmic increase of the damping force *F*_*b*_≈*b*_eff_*wA* (red dots) with *A* (that is, *wA*), which is attributed to the molecular pinning–depinning kinetics of the contact line. Here the normalization factors, *k*_0_ and *b*_0_*w*, are the initial values of *k*_eff_ and *b*_eff_*w*, respectively, obtained at *A*≡*A*_0_≈0.5 nm. Error bars indicate s.d. from four independent experiments.

To justify the proposed microscopic mechanism of the bridge-mediated noncontact friction, we have investigated the dependence of the interaction force on the shearing amplitude *A*. The tip first approaches to form the capillary nanobridge and during its subsequent retraction, the tip stops at a given position *z*=1 nm (or the bridge height 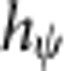
≈5.6 nm) for measurements of *k*_eff_ and *b*_eff_*w* while varying *A* (up to 30 nm). Figure 3 presents the behaviours of the force constants *k*_eff_ and *b*_eff_*w*, and the corresponding forces |*F*_*k*_| and |*F*_*b*_|, which are normalized respectively to *k*_0_ and *b*_0_*w* for convenience (here, *k*_0_ and *b*_0_*w* are the initial values of *k*_eff_ and *b*_eff_*w* obtained at *A=A*_0_≈0.5 nm). For the damping results, we used three fitting parameters *U*, *λ* and *α*, and our model reproduces excellently the measured *b*_eff_*w* (red curves in Fig. 3). On the other hand, for the elasticity part, we used no adjustable parameters, and the *k*_eff_ values show qualitative agreement although they are overall slightly larger than the results for the simple column model of the bridge (black curves in Fig. 3).

In Fig. 3a, we observe two characteristic behaviours of *k*_eff_ and *b*_eff_*w*. First, they both exhibit maximum values in the limit of small *A*. Second, the elastic coefficient decays faster than the damping constant with respect to *A*. These features are expected from the functional forms of equation (1) and equation (2), where both *F*_*k*_ and *F*_*b*_ initially increase linearly with *A*, but the force increments are suppressed as *A* increases. That is, while *F*_*k*_(≈*k*_eff_*A*) converges to a specific value as *A*→∞, *F*_*k*_(
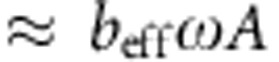
) increases logarithmically with *A*, so that *F*_*k*_/*A* is expected to decay faster than *F*_*b*_/*A*. We also notice that *b*_eff_*w* initially increases slightly from *A*=0.5 nm to below *A*=10 nm, in which the higher rate of depinning occurs at the larger *A*. Therefore, *α*(*A*) increases up to 1 for *A*<10 nm, but *α*(*A*)=1 remains constant beyond *A*=10 nm. Indeed, we fit excellently the experimental data *b*_eff_*w* by using *α*(*A*) such that *α*(*A*) is the linear curve connecting two points, *α*=0.6 at *A*=0.5 nm and *α*=1 at *A*=10 nm for *A*≤10 nm, whereas *α*(*A*)=1 for *A*>10 nm (red curves in Fig. 3).

Figure 3b presents the corresponding behaviours of the capillary shear forces versus *A* (or equivalently the shear velocity *wA*). Interestingly, the capillary shear friction increases logarithmically with the sliding velocity (see inset), similarly to the stick-slip contact friction in dry condition, despite the constant volume of the capillary bridge during measurements (Supplementary Note 7), which is realized due to the fast tip oscillation and the resulting short measurement time (that is, lock-in detection at *w*). This indicates that the logarithmic increase of the noncontact friction, resulting from the pinning–depinning ‘hopping' processes of the contact line, shares similar physical origins with the stick-slip mechanism of contact friction in dry condition. Interestingly, we observe this contact-line-induced friction may account for the recent solid–solid contact-sliding experiment on mica, where the logarithmic force increase was observed for constant normal load at low velocity^32^. In contrast, for sliding contact friction at relatively high velocity in ambient condition^11^, the force decreases logarithmically with velocity, which is attributed to the reduced friction as a result of the proportionately rapid diminishing of the capillary bridges due to evaporation of the contact-sliding meniscus and insufficient nucleation time for a meniscus formation^15^. Notice that at a farther tip–sample distance of *z*=5.2 nm, where the evaporation of the water bridge is more favourable so that the bridge becomes smaller, we observe the force starts decreasing soon after initial increase (Supplementary Note 7).

**Figure 4:**
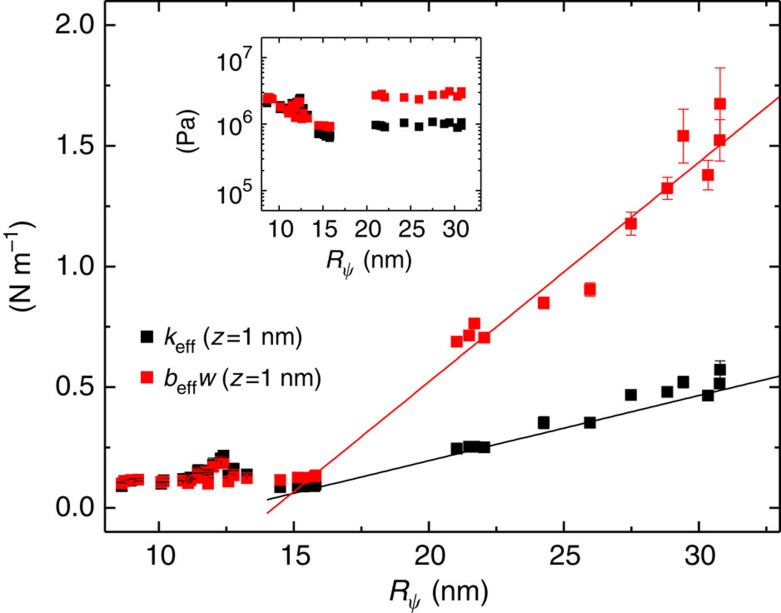
The contact-line radius 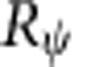 dependence of *k*_eff_ and *b*_eff_*w*. We measured *k*_eff_ (black squares) and *b*_eff_*w* (red squares) for small amplituide *A*<1 nm at *z*=1 nm while increasing RH from 2 to 60%, and plotted the force constants with respect to the corresponding 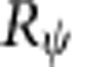, where 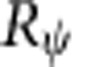 is numerically calculated based on the Young–Laplace equation^35^,^36^. The solid lines are the eye guides, showing the linear dependency on 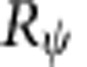 above 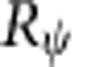
=15 nm, as predicted by equations (1) and (2). The inset presents the sizable elastic (black) and dissipative (red) shear modulus of the capillary-induced noncontact interaction, obtained from the measured *k*_eff_ and *b*_eff_*w*. Error bars represent s.d. for the measured values of *k*_eff_ and *b*_eff_*w* in the range of *z*=1±0.5 nm, which incorporates the maximum variation of ±0.5 nm in determination of the absolute capillary-condensation position relative to the sample at different RHs (refer to Supplementary Fig. 5).

Numerical calculation of the two intrinsic parameters, *λ* and *U*, indicates that the pinning–depinning processes of the contact line is associated with the dynamics at the molecular scale. In such a molecular process, *λ*, the mean distance between neighbouring pinning sites, should be an atomic-scale distance (<1 nm). Moreover, it has been argued by Blake and De Coninck that the activation energy per unit area is just the work of adhesion *W*_a_^33^, *U*/*λ*^2^≈W_a_, where 
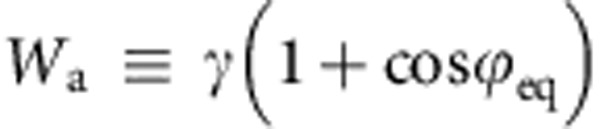
. Indeed, we obtain *λ*=0.6 nm and *U*=14.7*k*_*B*_*T* by fitting equation (2) to the experimental data for *A*>10 nm, which gives *U*/*λ*^2^=0.19 N m^−1^≈2*γ*≈*W*_a_, where *γ*=0.072 N m^−1^ at 20 °C. In addition, when depinning occurs, the force per unit length on the contact line is also expected to be the order of *U*/*λ*^2^, which is supported by equation (2), 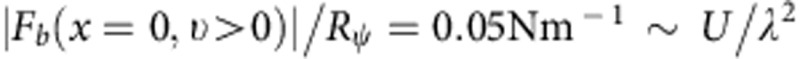. Consequently, the friction force *F*_*b*_ can be attributed as due to thermal activation of the water molecules at the contact line, being adsorbed and desorbed over the atomic scale *λ*=0.6 nm on the gold surface of the probe. It is interesting to observe that the sizable nonviscous damping that we observed even at *A*<1 nm already indicates the existence of the atomic-scale pinning–depinning processes (also refer Fig. 4).

Notice that water molecules near the contact line undergo incessantly repeating pinning–depinning (that is, adsorption–desorption or stick-slip) motion caused by the moving tip, which results in resistive frictional force. Such a friction at the molecular scale is manifested as the continuous and smooth energy dissipation at the macroscopic scale, described by the damping coefficient *b*_eff_, and it is responsible for the substantial energy dissipation that cannot be accounted for by usual hydrodynamic dissipation. A similar continuous macroscopic manifestation of the microscopic pinning–depinning processes as the energy dissipation has been shown in a different system that consists of a colloidal particle at the liquid interface^34^, where enhanced energy dissipation associated with the contact line was also observed beyond the predictions of the hydrodynamic viscous damping.

By varying RH, we observe that the noncontact sliding interaction can be enhanced by an order of magnitude with the increase of RH from 2 to 60%. Because the larger volume of the liquid bridge is formed at higher RH (Fig. 1d,e), the contact-line radius 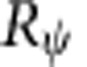 increases with RH for a fixed tip–sample distance. Specifically, by the numerical calculation based on the Young–Laplace equation^35^,^36^, we find 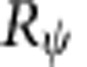 varies from 7 to 32 nm as RH increases from 2 to 60% at *z*=1 nm or 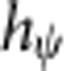
≈5.6 nm (see Supplementary Fig. 5 for details on a fine control of 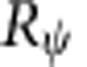). In Fig. 4, we present the interaction coefficients obtained at *z*=1 nm as a function of 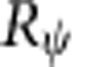, measured for small *A* (<1 nm), where *k*_eff_ and *b*_eff_*w* at RH=60% become 5 and 10 times larger than the values at RH=2%, respectively.

Figure 4 shows that *k*_eff_ and *b*_eff_*w* are linearly proportional to 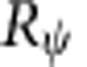 at 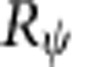
>15 nm. This linearity is expected because the forces, equations (1) and (2), are linearly dependent on 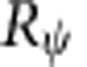. Notice that the linear behaviour holds above a certain value of 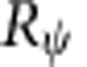, which is because the current model of the bridge-induced interaction does not take into account the detailed geometry of the water bridge. For example, for the real geometry of a bridge, there exists a critical value *R*_0_ for the minimum contact-line radius, at which the neck diameter of the bridge vanishes and thus *F*_*k*_=*F*_*b*_=0 at 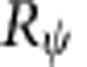
=*R*_0_, unlike equations (1) and (2) that predict the forces become zero only when 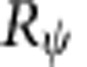
=0, which cannot hold below *R*_0_. Moreover, the continuum material model of the liquid bridge may break down for 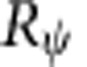
<15 nm, where the substantially differing behaviours of *k*_eff_ and *b*_eff_*w* are observed (Fig. 4), so that the discrete nature of the nanosized matter should be considered (see ref. 37).

## Discussion

For a unifying understanding, let us compare the bridge-mediated noncontact friction with the solid–solid contact friction, both of which possess the similar mechanism of the pinning–depinning or the stick-slip dynamics. The contact friction is characterized by the ‘interfacial shear strength', which is the friction divided by the real solid–solid contact area and is typically of the order of 10^8^ Pa^38^,^39^. For the present noncontact friction, we obtain the complex shear modulus G*, which is defined as the ratio of stress to strain and is represented by the complex sum of the storage modulus *G*′ and the loss modulus *G*″, that is, G*=*G*′+*G*″. While *G*′ represents the measure of the bridge resistance to being deformed elastically against the applied shear force, *G*″ shows the corresponding energy dissipation with respect to the same strain of the bridge. This leads to 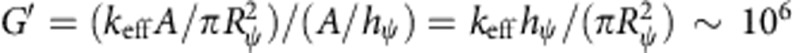 Pa and 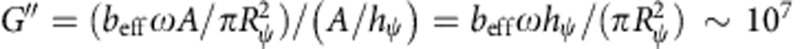 Pa, which are similar in magnitude to those of rubber (inset, Fig. 4). Notice that *G*″ is 10^4^ times larger than the bulk water, a substantial enhancement over the hydrodynamic effects between the tip and bridge. Interestingly, we emphasize that the noncontact shear dissipative modulus of a single bridge is up to 10% of the contact modulus, indicating that the noncontact friction may even dominate the contact friction depending on the conditions such as the number of capillary bridges.

In conclusion, we present the microscopic mechanism of the noncontact friction where the capillary shear interaction plays a key role. In particular, the novel nonviscous damping is attributed to the nonlinear pinning–depinning molecular processes of the tip–bridge contact line, which increases logarithmically with the shear velocity, as similarly observed in the atomic-scale ‘stick-slip' friction between solids in contact in dry condition. Notice that although the previous contact friction experiments in humid condition exhibit logarithmic decrease due to the reduced capillary-adhesion effect with velocity^16^, the present contact-line-induced friction may occur at the initial, low-velocity stage of the contact sliding where the capillary shear is nonnegligible. Our results offer a new mechanism of the nanotribology in ambient condition, which can be used for better microscopic understanding of both the macroscopic friction where numerous asperities exist and the rheological properties of the nanoscale interfacial fluids, and for designing and controlling the micro/nano electro-mechanical systems.

## Methods

### Experimental details

We used an etched gold tip and mica substrate in AFM operation, and they were prepared as follows: the gold tip was made by electrochemical etching in a 3.5-M HCl solution, which typically produced a tip diameter of 400∼500 nm. The tip was attached to one of two prongs of the quartz tuning fork. For the mica substrate, freshly cleaved mica was rinsed using dilute acid 5% (acetic acid) and then using deionized water. The tip and sample were placed in a homebuilt air-tight humidity-controlled metallic chamber at RH=2%, where they were dried for several hours before making measurements. The metallic chamber is equipped with two inlets for dry and H_2_O-saturated nitrogen gases and with one outlet connected to a vacuum pump. By varying the gas flows through the inlets, we control the relative humidity in the chamber. In addition, we also performed the experiment using a cantilever Si tip with a smaller radius of curvature, which produced a correspondingly smaller bridge and smaller force constants were measured. Further, when we used a hydrophobic, highly ordered pyrolytic graphite (HOPG) substrate instead of hydrophilic mica, the capillary-condensed water bridge was not produced, so that there is no bridge-mediated contribution to friction.

## 

## Additional information

**How to cite this article:** Lee, M. *et al*. Noncontact friction via capillary shear interaction at nanoscale. *Nat. Commun*. 6:7359 doi: 10.1038/ncomms8359 (2015).

## Supplementary Material

Supplementary Information Supplementary Figures 1-5, Supplementary Notes 1-7 and Supplementary References
