# Supplementary material for: Noncontact friction via capillary shear interaction at nanoscale
Source: Nat Commun. 2015 Jun 12;6:7359. doi: 10.1038/ncomms8359 (PMC4490357; doi:10.1038/ncomms8359)
Supplement: Supplementary Information — Supplementary Figures 1-5, Supplementary Notes 1-7 and Supplementary References [file ncomms8359-s1.pdf]

Supplementary Figures

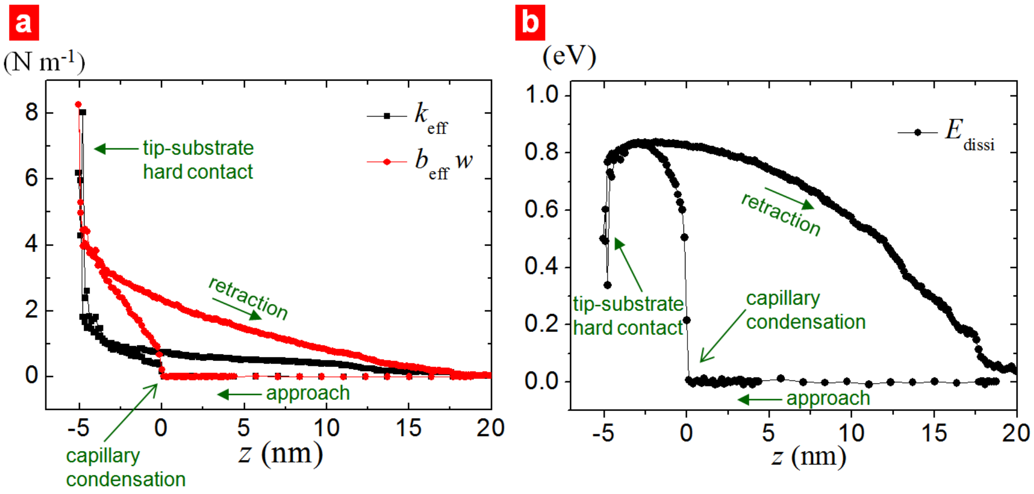

Supplementary Figure 1. Determination of the tip-sample contact point by the full approach-retraction curve. As the tip approaches the sample surface, the capillary-condensed water-bridge is formed at z=0, and then there occur simultaneously abrupt changes in (a) the elastic and damping coefficients, k_{eff} and $b_{\mathrm{eff}}w$, as well as (b) the energy dissipation E_{dissi} at about z=-4.6 nm, which indicates the tip-sample hard contact. This measurement was made at RH=61% after performing the entire experiments presented in the main text.

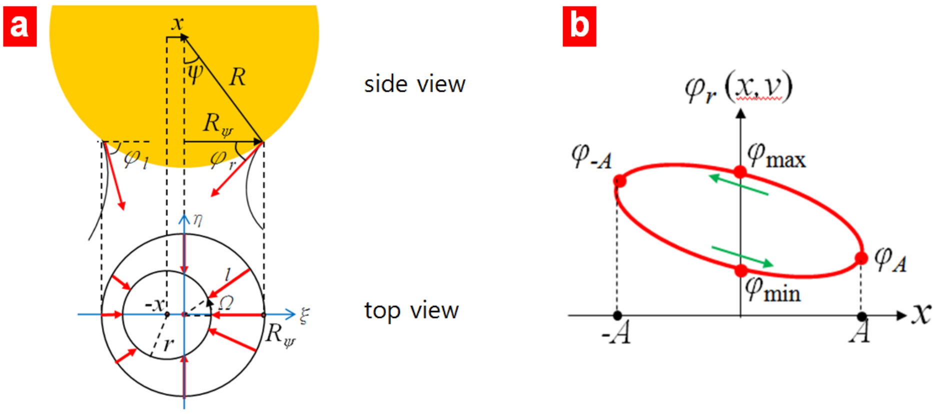

Supplementary Figure 2. Schematics of the contact-line-induced shear interaction. (a) The three-phase contact-line of the capillary liquid-bridge in air produces the tangential forces (red arrows). The net force then results in the restoring elastic force as well as the nonviscous damping force in the lateral direction under the shear motion of the tip. (b) Schematic diagram of the dynamically varying angle φ_{r} under the harmonically oscillating shear motion of the tip.

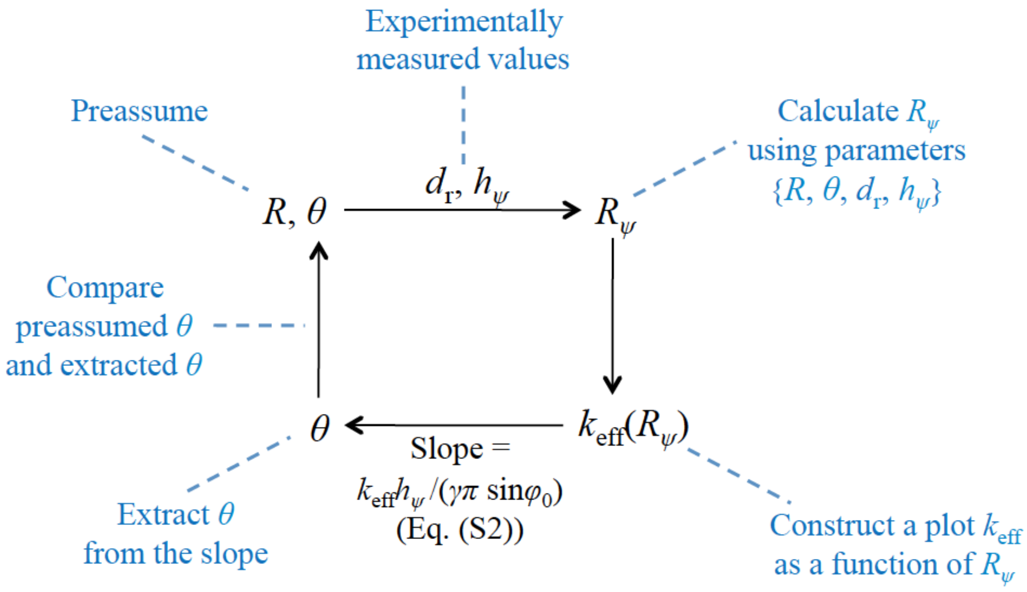

Supplementary Figure 3. An iterative method for obtaining the liquid-tip contact radius R_ψ.

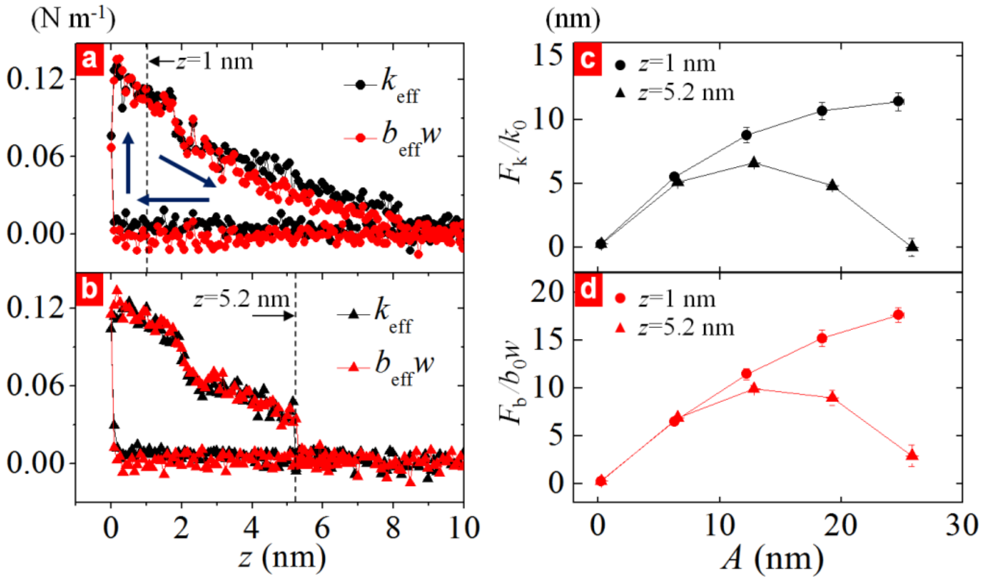

Supplementary Figure 4. Amplitude-dependence of F_k and F_b for two independent liquid bridges; (a) a bridge with constant volume sheared at z=1 nm and (b) a bridge with diminishing volume in time sheared at z=5.2 nm. (c,d) While both F_k and F_b at z= 1 nm increase with A (or velocity), they increase initially when sheared at z= 5.2 nm, but soon decrease with A due to the accordingly diminishing R_ψ. These characteristics are confirmed by Eqs. (1,2) in the main text.

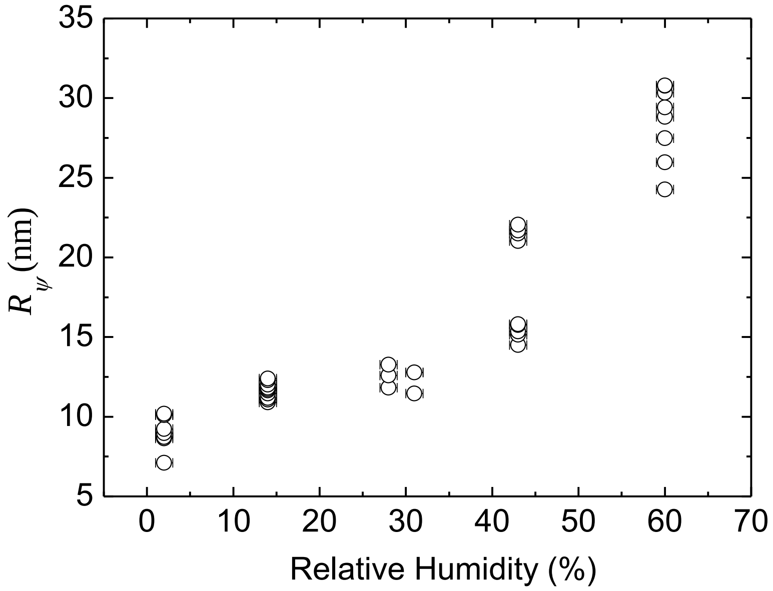

Supplementary Figure 5. Contract radius R_ψ versus relative humidity (RH). To observe the explicit dependence of R_ψ in Fig. 4 (main text), one should be able to vary R_ψ by small amounts. However, it is experimentally difficult to achieve fine variations of R_ψ only by changing RH; we actually realized slight increase of R_ψ by repeating the approach-retraction cycles at a given position on the substrate as well as at a given RH. In other words, as the water-bridge formation is performed between the tip and sample, some water molecules are left on the surfaces of tip and sample, which makes the water bridge that is obtained at the next approach-retraction cycle slightly bigger than the previous one due to the presence of the pre-existing water layer on the surfaces. As shown in the figure, while R_ψ shows an overall increase with RH, several closely-spaced values of R_ψ are obtained at a given RH. This feature was observed in all the experiments on RH dependence, especially prominent at high RH. Error bars indicate the maximum variation (±1%) of RH. It is important to notice that the same value of k_{eff} or $b_{\mathrm{eff}}w$ is observed for the identical value of R_ψ (Fig. 4, text) even if R_ψ can be obtained at different values of RH, which validates our experimental method. Notice also that measurements at different values of RH are made at different positions on the sample surface.

Supplementary Note 1

In ambient condition, water bridges are typically formed below ~10 nm separation. Therefore, between two macroscopic contacting surfaces with roughness of ~10 nm, most of the noncontact asperities will form the capillary-condensed bridges. On the other hand, for contact sliding between macroscopic surfaces with very large roughness, water bridges may not be easily formed between noncontact asperities. The actual distance between noncontact asperities depends on the weight of the macroscopic bodies themselves as well as the externally exerted normal force.

Supplementary Note 2

Determination of the tip-sample contact point

In our shear-mode AFM, we carefully define the tip-sample zero distance, based on the full approach-retraction curves of the elastic and damping coefficients, k_{eff} and $b_{\mathrm{eff}}w$, as well as the energy dissipation E_{dissi} [1]. Supplementary Figure 1(a) shows a full cycle of approach-retraction of k_{eff} and $b_{\mathrm{eff}}w$. As the tip approaches the sample surface, the capillary condensation first occurs at z=0 with the subsequent increase of k_{eff} and $b_{\mathrm{eff}}w$, the values of k_{eff} and $b_{\mathrm{eff}}w$ show abrupt increase at about z=-4.6 nm, and then the tip retracts from z=-5 nm to the original position. Interestingly, both k_{eff} and $b_{\mathrm{eff}}w$ increase rapidly at z=-4.6 nm during approach, whereas the energy dissipation E_{dissi} first increases, but then decreases suddenly near the same position of z=-4.6 nm, as shown in Supplementary Figure 1(b).

This abrupt decrease of E_{dissi} indicates that the tip-substrate interaction becomes increasingly elastic and thus energy dissipation becomes negligible, which occurs when the tip-sample contact is made. In our analysis of experimental results, therefore, we have set the contact point at z=-4.6nm, where k_{eff} and $b_{\mathrm{eff}}w$ abruptly increase and E_{dissi} rapidly decreases. We find that this definition of the contact point is proper enough to describe the geometry of the capillary bridge formed in the tip-sample gap and the associated interaction forces. Moreover, we emphasize that the experimental results vary only within less than 20% variation (z=-4.6±1 nm) in the contact point and thus the main conclusions made are not affected. Notice that there have been reports on the capillary condensation of the water nanobridges at the distance far beyond the Kelvin radius, similar to our case (see for example, [2]).

Supplementary Note 3

Determination of the geometrical quantity R_ψ of the capillary liquid-bridge

To obtain the contact-line radius R_ψ (Supplementary Figure 2(a)) at a specific tip-sample distance (for example, z=1 nm or $h_\psi \approx$ 5.6 nm as in the case of Fig. 4 of the main text), which is an important parameter as shown in Supplementary Equations (1-3), we use the theoretical formalism described in ref. [3], based on the Young-Laplace equation. This method allows to determine the detailed geometrical parameter of a liquid bridge such as R_ψ using the boundary condition of constant volume, for the given tip's radius of curvature R, tip-bridge contact angle θ, substrate-bridge contact angle θ', and volume of the liquid bridge V_0. Here the contact angle θ is obtained as $\theta = \varphi_0 - \psi$ where φ_0 is the equilibrium angle at x=0 (see Supplementary Figure 2(a)). In addition, we use Eq. (14) in ref. [4] to obtain V_0 from the experimentally measured rupture distance d_{r} (measured with respect to the contact point; see Supplementary Note 2) under the assumption $\theta'=\theta$. Therefore, once R, θ and d_{r} are known, R_ψ at a specific distance h_ψ can be uniquely determined by this formalism.

Supplementary Figure 3 presents the flow diagram that details the iterative derivation of the liquid-tip contact radius R_ψ. Although d_{r} and h_ψ can be directly measured by experiment, it is difficult to know a priori the values of R and θ experimentally. Therefore, to calculate R_ψ with unknown R and θ, we use the following iterative procedures: we (i) first pre-assume the values of R and θ, (ii) calculate R_ψ by using experimentally measured d_{r} and h_ψ, (iii) extract θ from the slope of the linear fit for $k_{\mathrm{eff}}(R_\psi)$, which is given by $k_{\mathrm{eff}}h_\psi$ /($\gamma\pi$ sinφ_0) (using Supplementary Equation (2) and $\kappa_k \approx 1/h_\psi$ as shown in Supplementary Note 5), and then (iv)

compare the extracted θ to the pre-assumed θ to find out the self-consistent values of R and θ, which allows the desired value of R_ψ. By this method, we obtained R=400 nm and θ =33˚. As a result, once R_ψ is known, we can plot k_{eff} as a function of R_ψ, as shown in Fig. 4 of the main text. Notice that we also confirmed the linearity of k_{eff} and $b_{eff}w$ (in Fig. 4) is unaffected for 12 different combinations of parameters; R=300, 400, 500 nm, infinity (flat) and θ=23, 33, 43°, while only about 10% variation occurs in the calculated absolute values of R_ψ.

Supplementary Note 4

Estimation of the hydrodynamic interaction on the tip-liquid contact interface

Hydrodynamic interaction between the tip surface and the liquid bridge is associated with the fluid dynamics within the confined liquid and the corresponding shear stress exerted on the tip surface. The resulting interaction is estimated by the sum of the damping forces arising from (i) the Couette flow in the most area of the tip-liquid interface and (ii) the viscous flow within the wedge of the liquid near the moving contact-line. The hydrodynamic damping of (i) is well described by the formula, $w\mu\sigma/h_\psi$, presented in the main text, for the fluid system of low Reynolds number ($\equiv\rho wAh_\psi/\mu \sim 10^{-6}$). This interaction results in a damping coefficient of about 10^{-5} N/m, much smaller than the observed value of 1 N/m, as discussed in the main text. On the other hand, the viscous interaction of (ii) occurring in the proximity to the contact-line originates from the bending of the liquid/vapor interface, resulting in the change in the contact angle. The contact-angle change is estimated to be $\varphi^3-\varphi_{eq}^3=9\ Ca\ l$ [5], as shown in the main text. Here $l=\log(l_m/l_0)$ is the ratio of macroscopic to microscopic length scales, which typically falls into the range of 1~100. And Ca is the capillary number defined by $\mu wA/\gamma$, where μ is the viscosity of liquid, w the tip's oscillation frequency, A the oscillation amplitude and γ the surface tension. Then, we obtain a relation between the angle change and the velocity, given by $\Delta\varphi/A=\mu wl/(3\gamma\varphi_{eq}^2)$. By using Supplementary Equation (3), and also employing the numerically obtained parameters for our tip-fluid system described in Supplementary Note 3, we can finally estimate the damping coefficient as $\sim 10^{-4}$ N/m for R_ψ=15 nm and l=100, which is 10^4 times smaller than the observed damping ~1 N/m. Therefore, we conclude that the hydrodynamic interaction cannot address the observed enhanced damping as well as the elastic forces.

Supplementary Note 5

Theory of the contact-line-induced shear interaction

To derive the capillary 'shear' interaction originating from the three-phase contact-line on the tip-bridge interface, we discuss how the tip-liquid angle φ is changed as the tip (radius of curvature R) moves laterally (Supplementary Figure 2(a)). When the tip moves to the right in the x direction, the shape of the liquid bridge capillary-condensed in the tip-substrate nano gap is slanted, changing the tip-liquid angle $\varphi(\Omega)$. We define $\varphi(\Omega=0)\equiv\varphi_r$ (i.e., the angle at the very right end in the side view) and $\varphi(\Omega=\pi)\equiv\varphi_l$ (i.e., the angle at the very left end). In general, $\varphi(\Omega)$ depends on the tip surface-liquid wetting property as well as the tip's motion. For example, if the tip is super-hydrophilic (super-hydrophobic), then $\varphi(\Omega)=\psi$ ($\varphi(\Omega)=\psi+\pi$) by definition. Although one can derive $\varphi(\Omega)$ by considering the three-dimensional shape of the slanted liquid bridge for a complete description, we assume a circular distribution of $\varphi(\Omega)$ for

small displacement x to obtain the analytical form of the interaction, as described in Supplementary Figure 2(a).

Here the inner circle with a radius r in the ξ-η plane (the blue-colored coordinate frame in Supplementary Figure 2(a)) determines the magnitude of the horizontal component of the tangential force f in such a way that the force is proportional to the physical length of the red arrow l; $f(\Omega)= \tau\, l(\Omega)$, where τ is a constant. Then, the two unknowns r and τ can be exactly determined by the two known relations, $f(0)=\gamma\cos\varphi_r$ and $f(\pi)=\gamma\cos\varphi_l$, where γ is the surface tension of the liquid. By integrating $f(\Omega)$ over $0 \le \Omega < 2\pi$, we obtain the contact-line-induced force exerted by the capillary liquid-bridge,

$$F_{\text{line}} = -\frac{\pi\gamma R_\psi}{2}(\cos\varphi_r - \cos\varphi_l). \qquad \text{(Supplementary Equation 1)}$$

Supplementary Equation (1) describes the instantaneous value of the capillary shear force in terms of the two contact angles $\varphi_r \equiv \varphi(\Omega=0)$ and $\varphi_l \equiv \varphi(\Omega=\pi)$, which generally depend on the tip displacement x and velocity v, so that $\varphi_r=\varphi_r(x,v)$, as schematically presented in Supplementary Figure 2(b). In order to investigate the quantitative characteristics of the shear force, we adopt a simple form of $\varphi_r=\varphi_r(x,v)$ as an ellipse that connects smoothly the four fixed points φ_{-A}, φ_A, $\varphi_{\max}$, and $\varphi_{\min}$. As a result, a functional form of F_{line} can be obtained, from which we can derive the elastic and damping constants of the shear interaction using the relations, $k_{\text{eff}}=F_k/A$ and $b_{\text{eff}}w=F_b/A$,

$$k_{\text{eff}} = \pi\gamma\kappa_k R_\psi \sin\varphi_0, \qquad \text{(Supplementary Equation 2)}$$

$$b_{\text{eff}}\, w = \pi\gamma\kappa_b R_\psi \sin\varphi_0. \qquad \text{(Supplementary Equation 3)}$$

Here $\kappa_k \equiv (\varphi_{-A}-\varphi_A)/(2A)$ is the slope of the fitted ellipse, $\kappa_b \equiv (\varphi_{\max}-\varphi_{\min})/(2A)$ characterizes the hysteresis at $x=0$, φ_0 is the equilibrium angle at $x=0$, and R_ψ is the contact-line radius (Supplementary Figure 2(a)).

Although Supplementary Equations (2,3) formally describe k_{eff} and b_{eff} in terms of the angle changes κ_k and κ_b, respectively, this mechanistic description alone does not predict the detailed values of κ_k and κ_b for a given shear motion of the tip. First, for quantitative investigation of κ_k, we consider simply an ideal cylindrical shape of the liquid bridge. When shearing the liquid column ($\varphi_0=\pi/2$), we can obtain analytically the angle change under small oscillation as follows, $\kappa_k \equiv (\varphi_{-A}-\varphi_A)/(2A)=2(\pi/2-\varphi_A)/2A \approx \tan(\pi/2-\varphi_A)/A=1/h_\psi$. Using this approximation, we find $k_{\text{eff}}=\pi\gamma\,(1/h_\psi)R_\psi$ and this is exactly the leading term of k_{eff} derived from Eq. (1) in the main text, which is based on the explicit calculation of the interfacial energy change for the cylindrical bridge (see the text below Eq. (1) in the main text).

For the damping coefficient, on the other hand, we consider the 'hopping' dynamics of the capillary contact-line, as discussed in the main text. For detailed derivation of the damping constant, one has to sum over the externally imposed forces per unit length on the contact-line, $f_l=\gamma(\cos\varphi_{eq}-\cos\varphi_d)$, and the explicit summation results in the analytic expression that is proportional to the term, $(\cos\varphi_r-\cos\varphi_l)$. Notice that Supplementary Equation (1) connects this

term, $(\cos\varphi_r – \cos\varphi_l)$, to the total shear force F_{line}, which is reduced to F_{b} for x=0 (or v=wA). Furthermore, the 'hopping' model relates the damping force $F_{\mathrm{b}}(x=0, v=wA)$ with the contact-line velocity V, which is proportional to the tip's shear velocity wA (refer to the main text for details). Following these steps, we finally obtain Eq. (2) in the main text.

Supplementary Note 6

Derivation of the surface-energy change U_l for a sheared cylindrical column

Here we present detailed derivation of the surface-energy change U_l for the simple cylindrical column of water (see inset of Fig. 2(a) in the main text). Consider the top circular surface of the cylindrical column whose radius is R_ψ. Under the shear displacement of x along the x-axis, the incremental surface area dS of the side wall of the cylinder is given by, $\mathrm{d}S = \text{base} \times \text{hypotenuse} = R_\psi \mathrm{d}\zeta \times \sqrt{x^2\cos^2\zeta + h_\psi^2}$ where ζ is the polar angle with respect to the x-axis and h_ψ is the height of the sheared cylinder. The corresponding increase of the surface energy for a given x, dU_l, is then written as $\mathrm{d}U_l = \gamma \mathrm{d}S$ where γ is the surface tension of water. Therefore, the resulting total increase of the surface energy U_l is given by,

$$U_l = \int \mathrm{d}U_l = 4\int_0^{\pi/2} \gamma R_\psi h_\psi \sqrt{\left(1-\left(ix/h_\psi\right)^2\cos^2\zeta\right)}\, d\zeta, \qquad \text{(Supplementary Equation 4)}$$

which can be rewritten as,

$$U_l = 4\gamma R_\psi h_\psi E\left[\left(ix/h_\psi\right)^2\right]. \qquad \text{(Supplementary Equation 5)}$$

Here the function E is the complete elliptic integral of the second kind. Notice that the total increase of the surface area S for a given x is $S = 4\int_0^{\pi/2}\mathrm{d}S = 4R_\psi h_\psi E\left[\left(ix/h_\psi\right)^2\right]$.

Supplementary Note 7

Shear-amplitude dependence of k_{eff} and $b_{\mathrm{eff}}w$ for a liquid bridge with decreasing R_ψ

In the main text, we have presented the shear-amplitude dependence of k_{eff} and $b_{\mathrm{eff}}w$ for a liquid bridge with the constant volume or constant R_ψ (Fig. 3 in the main text). We emphasize this condition of constant volume is confirmed by the fact that the interaction force measured after the amplitude-dependence experiment done at a given RH is fully recovered to the initial value of the force (Supplementary Figure 4(a)). Under this condition of constant bridge volume, we have observed the logarithmically increasing shear frictional force with the increase the shear amplitude (or velocity), as shown in the inset of Fig. 3(b) and also predicted by Eq. (2) in the main text.

Now let us consider the noncontact sliding friction mediated by a liquid bridge whose volume decreases in time during the shear motion. This diminishing bridge volume leads to

the decreasing R_ψ during the friction measurement, as typically observed in the contact friction experiments that employ a linearly moving cantilever, which is due to the finite nucleation or growth time of the bridge [6]. Interestingly, in our experiment, the similar decrease of R_ψ is observed in shear measurement for the liquid bridge elongated up to z=5.2 nm, much longer than that in Fig. 3 of the main text. For this longer bridge, the evaporation process of the bridge is more favorable and thus R_ψ decreases rapidly with the shear motion.

Supplementary Figure 4(c,d) shows the experimentally measured k_{eff} and $b_{\text{eff}}w$ for a capillary bridge whose R_ψ decreases with the increase of A, i.e., for the bridge sheared at z=5.2 nm. As expected, we find that both the elastic and damping forces start increasing following the curves similar to those for the bridge sheared at z=1 nm, but then both decrease rapidly due to the diminishing R_ψ despite the increase of A. In fact, we observed no remaining shear forces when measured after the amplitude-dependence experiments were performed, unlike the z=1 nm case, which indicates that the liquid bridge indeed vanished during shear motions (Supplementary Figure 4(b)). In other words, the friction initially increases due to the dominant pinning-depinning dynamics of the contact-line, but then diminishes because of the reducing volume or R_ψ (see Eq. (2) in the main text).

Supplementary References

[1] In dynamic force spectroscopy, the energy dissipation E_{dissi} per one shear-oscillation cycle is given by $\pi A^2 b_{\text{eff}} w$, where A is the tip-oscillation amplitude.

[2] Greiner, C., Felts, J.R., Dai, Z., King, W. P. and Carpick, R. W. Controlling nanoscale friction through the competition between capillary adsorption and thermally-activated sliding. *ACS Nano* **6**, 4305–13 (2012).

[3] Stifter, T., Marti, O. & Bhushan, B. Theoretical investigation of the distance dependence of capillary and van der Waals forces in scanning force microscopy. *Phys. Rev. B.* **62**, 13667-13673 (2000).

[4] Willett, C. D., Adams, M. J., Johnson, S. A. & Seville, J. P. K. Capillary bridges between two spherical bodies. *Langmuir* **16**, 9396-9405 (2000).

[5] Cox, R. G. The dynamics of the spreading of liquids on a solid surface. *J. Fluid Mech.* **168**, 169–194 (1986).

[6] Szoszkiewicz, Robert & Riedo, Elisa. Nucleation time of nanoscale water bridges. *Phys. Rev. Lett.* **95**, 135502 (2005).
